# Glycation of macrophages induces expression of pro-inflammatory cytokines and reduces phagocytic efficiency

**DOI:** 10.18632/aging.102123

**Published:** 2019-07-29

**Authors:** Veronika Bezold, Philip Rosenstock, Jonas Scheffler, Henriette Geyer, Rüdiger Horstkorte, Kaya Bork

**Affiliations:** 1 Institute for Physiological Chemistry, Martin-Luther-University Halle-Wittenberg, Halle (Saale), Germany; 2 Octapharma Biopharmaceuticals GmbH, Molecular Biochemistry, Berlin, Germany

**Keywords:** aging, glycation, advanced glycation end products, macrophages, inflammation, methylglyoxal

## Abstract

Glycation and the accumulation of advanced glycation end products (AGEs) are known to occur during normal aging but also in the progression of several diseases, such as diabetes. Diabetes type II and aging both lead to impaired wound healing. It has been demonstrated that macrophages play an important role in impaired wound healing, however, the underlying causes remain unknown. Elevated blood glucose levels as well as elevated methylglyoxal (MGO) levels in diabetic patients result in glycation and increase of AGEs. We used MGO to investigate the influence of glycation and AGEs on macrophages. We could show that glycation, but not treatment with AGE-modified serum proteins, increased expression of pro-inflammatory cytokines interleukin 1β (IL-1β) and IL-8 but also affected IL-10 and TNF-α expression, resulting in increased inflammation. At the same time, glycation reduced phagocytic efficiency and led to impaired clearance rates of invading microbes and cellular debris. Our data suggest that glycation contributes to changes of macrophage activity and cytokine expression and therefore could support the understanding of disturbed wound healing during aging and diabetes.

## Introduction

Glycation and the accumulation of so called advanced glycation end products (AGEs) have been known to occur during normal aging [1] but also in the progression of several diseases, such as diabetes [2,3], Alzheimer’s disease [4,5], multiple sclerosis [6], and atherosclerosis [7]. Glycation is the non-enzymatic reaction of the carbonyl group of a reducing sugar (e.g. glucose or fructose) with a free amino group of a protein, forming a non-stable Schiff base [8]. Further rearrangement leads to formation of a more stable ketosamine, the so-called Amadori product. Irreversible modification of this Amadori product finally results in the formation of AGEs. In addition to sugars, highly reactive dicarbonyl compounds, such as methylglyoxal (MGO) or glyoxal, can also form AGEs [9]. MGO is a naturally occurring byproduct of glycolysis. Up to 0.4% of glucose molecules per cycle are metabolized to MGO [10,11]. During progression of several diseases as well as during aging, MGO concentrations are elevated [12]. AGEs can be recognized by several receptors on the cell surface. One of the best known is the receptor for advanced glycation end products (RAGE), a member of the immunoglobulin superfamily and a class J scavenger receptor [13,14]. Binding of AGEs to RAGE leads to cellular activation and internalization of the bound AGE structures [15], resulting in production of reactive oxygen species (ROS), induction of p44/p42 mitogen activated protein kinase and nuclear transcription factor κB (NF-κB) [16]. Homeostasis is maintained between produced and incorporated AGEs and their cleavage. However, this process can be altered for example by permanently higher blood glucose levels, that in turn lead to higher levels of AGEs [17]. With increasing age of the population, cardiovascular and metabolic diseases and impairments related to accumulation of AGEs are rising [18,19], as well as the incidence of type II diabetes [20,21]. Regarding diabetes, it has been shown that the plasma concentrations of MGO are increased two- to ﬁvefold compared to healthy individuals [22], indicating increased formation of AGEs [23]. It is also known that 4 - 10% of diabetic patients develop foot ulcers [24,25], resulting in infected wounds that display a chronic, pro-inflammatory phenotype [26], as well as impaired and delayed wound healing [27,28]. However, the underlying mechanisms of impaired wound healing in both diabetic and elder patients remain unknown and effective treatments are proving elusive.

**Figure 1:**
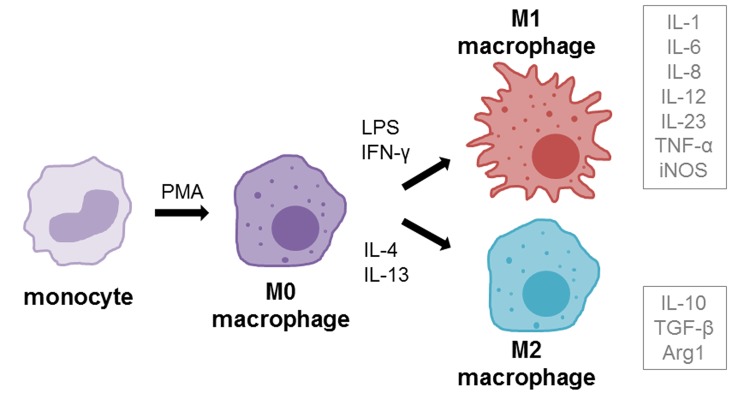
**Macrophage differentiation and polarization.** Monocytes can be differentiated into macrophages (resting, M0) using the differentiation agent 12-myristate 13-acetate (PMA). M0 macrophages can be further polarized into M1 (pro-inflammatory, classically activated) phenotype using LPS and IFN-γ or into M2 (anti-inflammatory, alternatively activated) using IL-4 and IL-13 treatment. Grey boxes beside polarization phenotypes show the cytokines that are mainly secreted by respective phenotype.

Macrophages play an important role in impaired wound healing, especially in diabetes [26,29,30]. Under normal conditions, monocyte derived macrophages (M0, resting) are able to polarize into M1 (classically activated) or M2 (alternatively activated) phenotypes when recruited into wounds [31,32] (Figure 1). M1 macrophages display pro-inflammatory functions, whereas the M2 phenotype reduces inflammation, induces tissue remodeling and plays a more regulatory role [31,33,34]. Resident macrophages in type II diabetic wounds tend to remain predominantly in the M1 activation state, leading to chronic inflammation [29,30,35]. Increased levels of M1 macrophages could be found during remodeling phase of wounds in a diabetic mouse model, whereas the population of M2 macrophages was very low [36]. Increased concentrations of pro-inflammatory cytokine interleukin-1 beta (IL-1β) could also be found in diabetic wounds and inhibition of IL-1β pathway resulted in improved wound healing via induction of a reparative macrophage phenotype [37].

Elevated MGO levels could already be detected in murine macrophages after infection with mycobacteria, affecting activation, immunity and apoptosis [38]. Also, it has been demonstrated that MGO treatment induces activation of murine macrophages and ROS production in Sarcoma-180 bearing tumor mice [39,40], and therefore can even be beneficial for the immune system as immunomodulation against tumors. In recent studies, we could demonstrate that MGO treatment had a negative effect on the activation of human natural killer cells [41]. In the present study, we used MGO as a glycating agent and analyzed inflammatory and functional properties of human macrophages after glycation, in comparison to treatment with AGE-modified serum proteins. We could show that glycation modulated cytokine expression and also altered phagocytic efficiency of macrophages, while treatment with soluble AGEs had no effects.

## RESULTS

### MGO induces glycation of THP-1 macrophages

**Figure 2:**
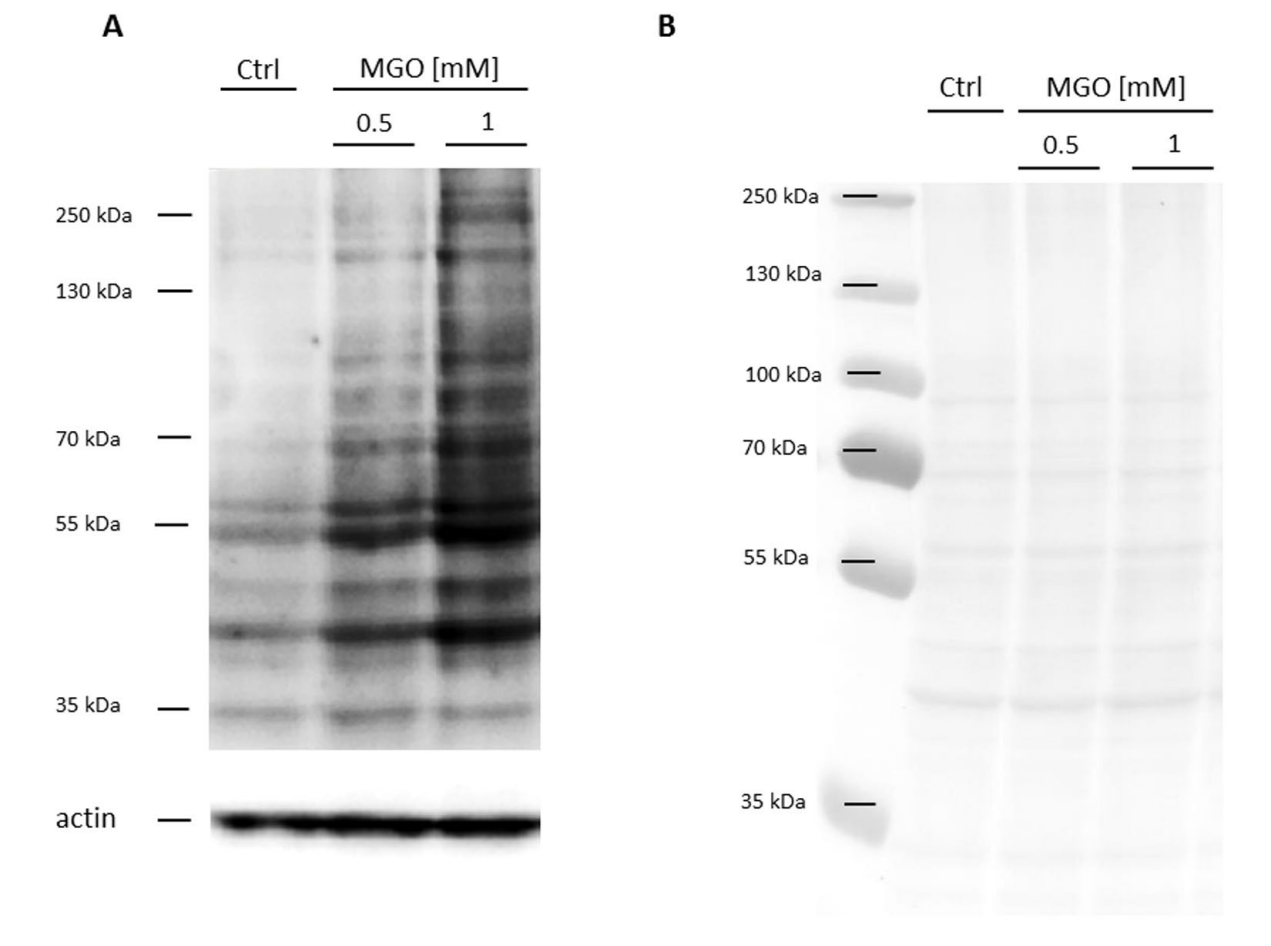
**Detection of AGE formation after glycation.** THP-1 macrophages (M0) were incubated with different concentrations of MGO for 24 h. Total proteins were separated by SDS-PAGE and immunoblotted. (**A**) Formation of AGEs was detected using an anti-AGE antibody (CML-26). The depicted blot represents 3 independent experiments. (**B**) Corresponding Ponceau staining of representative blot.

**Figure 3:**
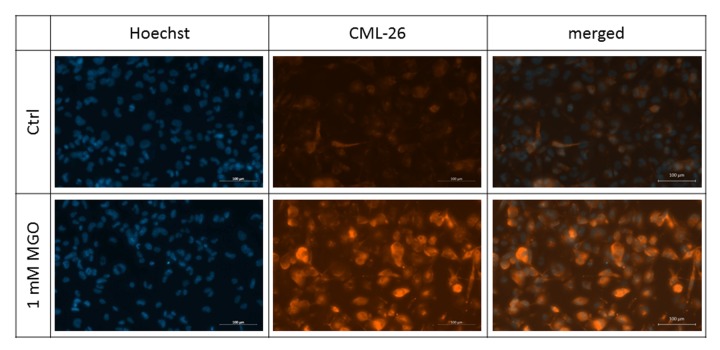
**Detection of surface glycation.** THP-1 macrophages (M0) were treated with or without 1 mM MGO for 24 h. Immunofluorescence staining of surface glycation was performed using an anti-AGE antibody (CML-26, shown in orange). Hoechst was used as nuclear stain (shown in blue). Shown pictures are representative for 4 independent experiments. Scale bars indicate 100 µm.

In the first series of experiments, we wanted to analyze whether MGO is able to induce glycation in macrophages. For this, THP-1 M0 macrophages were cultivated in the absence or presence of 0.5 or 1 mM MGO for 24 h. We analyzed the cells after MGO treatment using bright-field microscopy. We could not detect morphological changes between cultures grown in the absence or presence of MGO (Supplementary Figure 1). We then wanted to verify that MGO treatment induces cellular glycation of macrophages. We therefore isolated total protein from THP-1 macrophages, performed immunoblotting with an anti-AGE antibody (Figure 2) and found that MGO treatment leads to elevated cellular formation of AGEs, resulting in broad smear bands. Increasing MGO concentrations lead to increased band intensities, indicating increased formation of AGEs. Next we wanted to prove that MGO increases glycation of cell surface proteins. Therefore, we treated THP-1 macrophages with 1 mM MGO and performed immunofluorescence staining with an anti-AGE antibody (CML-26, Figure 3) without permeabilization of the cell membrane. We could demonstrate that, in comparison to the untreated control, MGO treated cells displayed a stronger AGE-dependent fluorescence signal on their surface, indicating a strong glycation of cell surface proteins.

### RAGE expression is not increased after glycation

**Figure 4:**
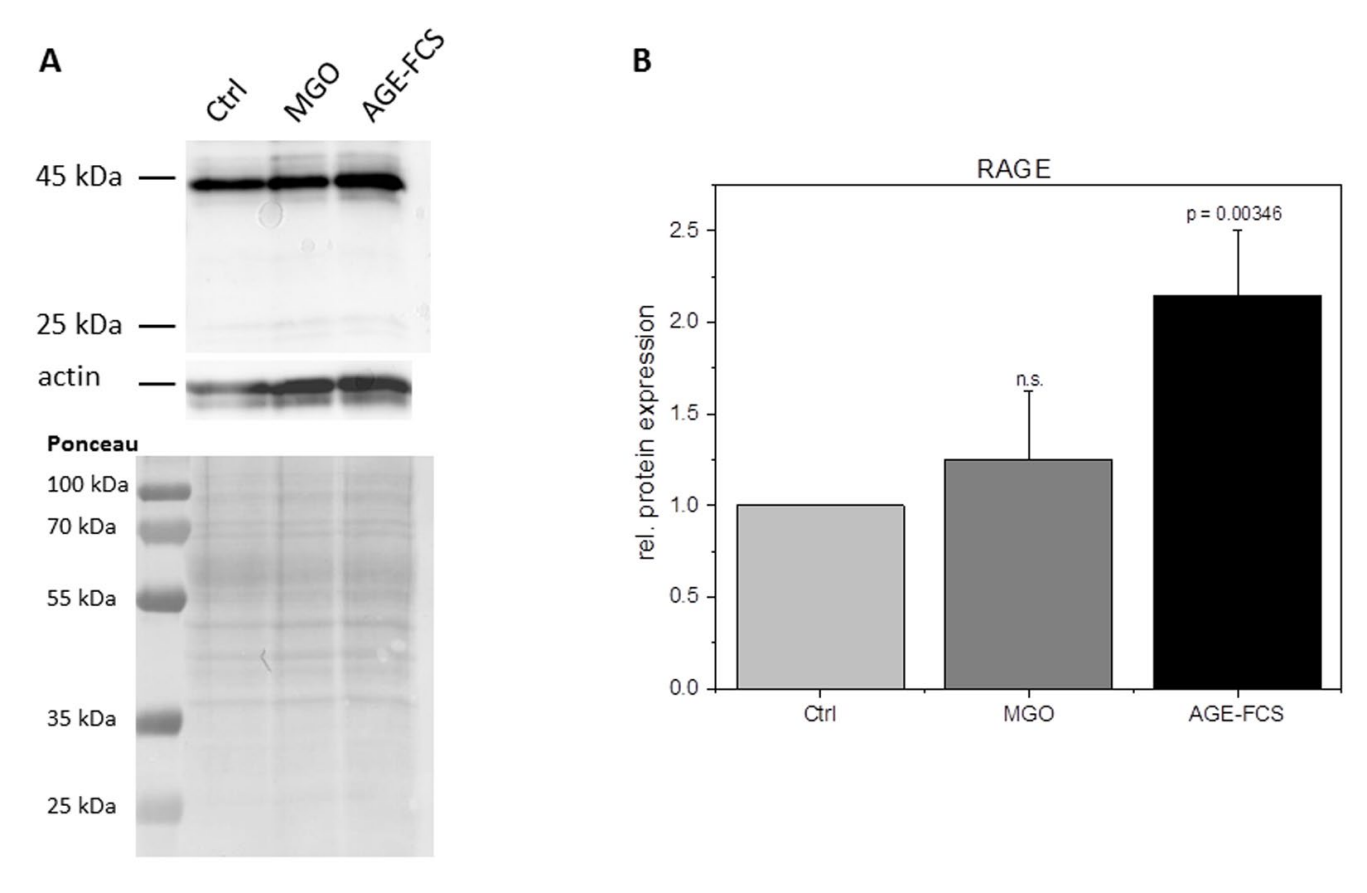
**RAGE expression after glycation.** THP-1 macrophages (M0) were incubated with 1 mM MGO or 10% AGE-FCS for 24 h in normal growth medium. Total protein was separated by SDS-PAGE and immunoblotting. RAGE expression was detected using an anti-RAGE antibody (ab3611); (**A**) and quantified in relation to actin staining (**B**). The Graph shows average mean of relative RAGE expression + SD of 4 independent experiments.

Glycation is known to increase expression of RAGE in many cells. In order to analyze RAGE in glycated THP-1 macrophages, we isolated total protein of M0 cells incubated with 1 mM MGO or medium containing 10% AGE-FCS. We then performed immunoblotting with an anti-RAGE antibody and quantified the band intensity in relation to actin staining (Figure 4). Glycation with 1 mM MGO did not upregulate RAGE protein expression, while treatment with soluble AGE-FCS leads to a more than two-fold increased expression of RAGE. To verify this, we also performed flow cytometry analysis of living M0 macrophages that were treated with 1 mM MGO or medium containing 10% AGE-FCS. For staining we used the same anti-RAGE antibody as mentioned above and a secondary FITC-labeled antibody (Supplementary Figure 2). We could show an increase of RAGE signal after treatment with AGE-FCS, but not after glycation of the cells with MGO, confirming our immunoblot data.

### Apoptosis is only induced at high MGO concentrations

**Figure 5:**
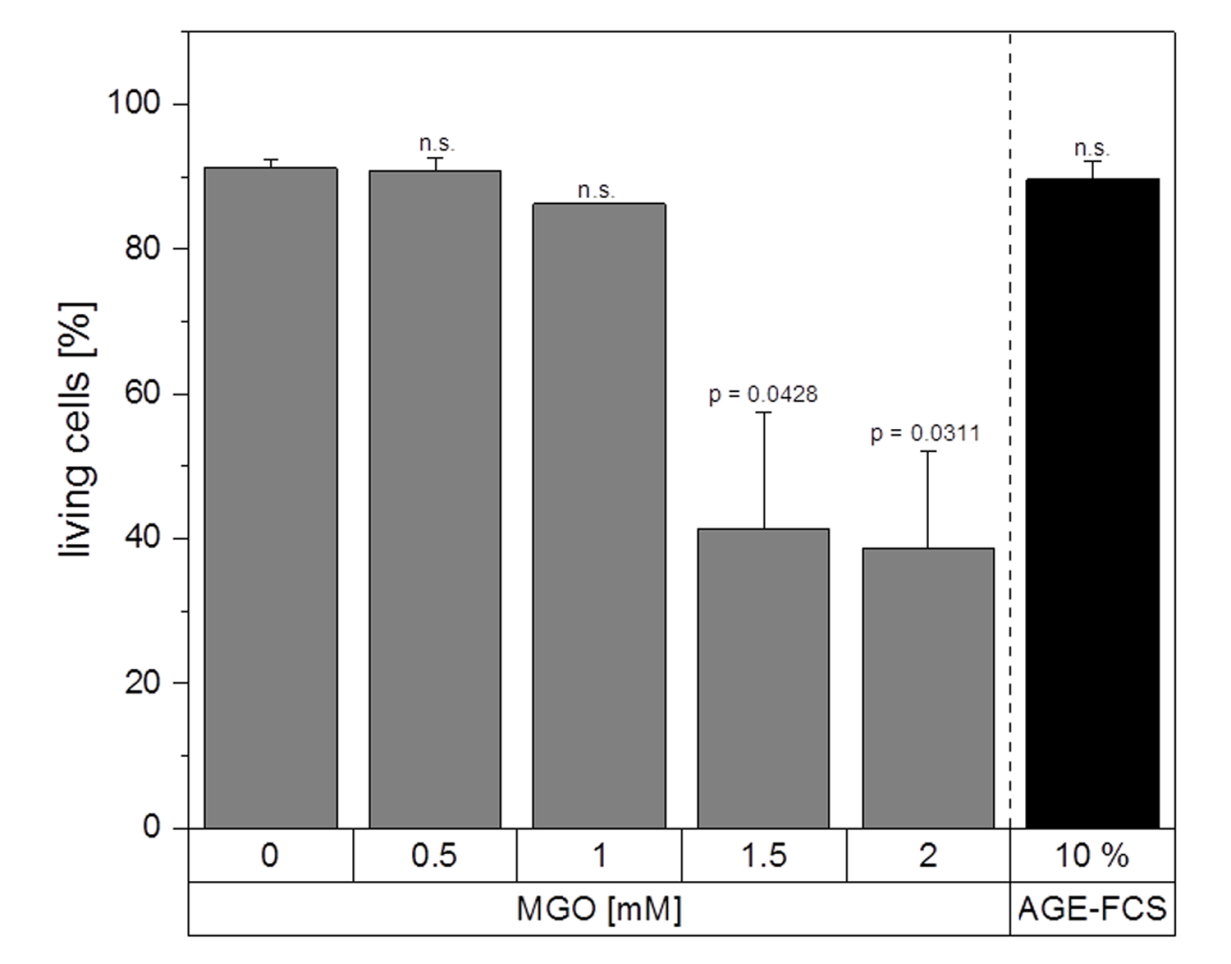
**Apoptosis assay.** THP-1 macrophages (M0) were treated with different MGO concentrations or 10% AGE-FCS for 24 h and apoptosis assay was performed using 7AAD and Annexin V staining. The percentage of Annexin V^-^ / 7AAD^-^ cells was used to determine the intact living cells (= non-apoptotic and non-necrotic). Graph shows average mean + SD of 3 independent experiments.

Next, we wanted to investigate whether MGO treatment leads to induction of apoptosis. Therefore, we performed apoptosis assays using Annexin V and 7AAD staining with macrophages treated with 0.5, 1, 1.5 and 2 mM MGO or 10% AGE-FCS for 24 h (Figure 5). Apoptosis was only induced using 1.5 and 2 mM MGO. All other tested MGO concentrations as well as treatment with AGE-FCS did not result in induction of apoptosis or necrosis. We also analyzed whether MGO treatment leads to a reduction of the metabolic activity of macrophages. Therefore, MTT assays were performed with macrophages treated with 0.5, 1, 1.5 and 2 mM MGO for 24 h (Supplementary Figure 3). We could demonstrate that MGO did not significantly reduce metabolic activity up to concentrations of 1 mM. However, 1.5 and 2 mM MGO led to a significant reduction of the metabolic activity.

### ROS production is not altered upon glycation

**Figure 6:**
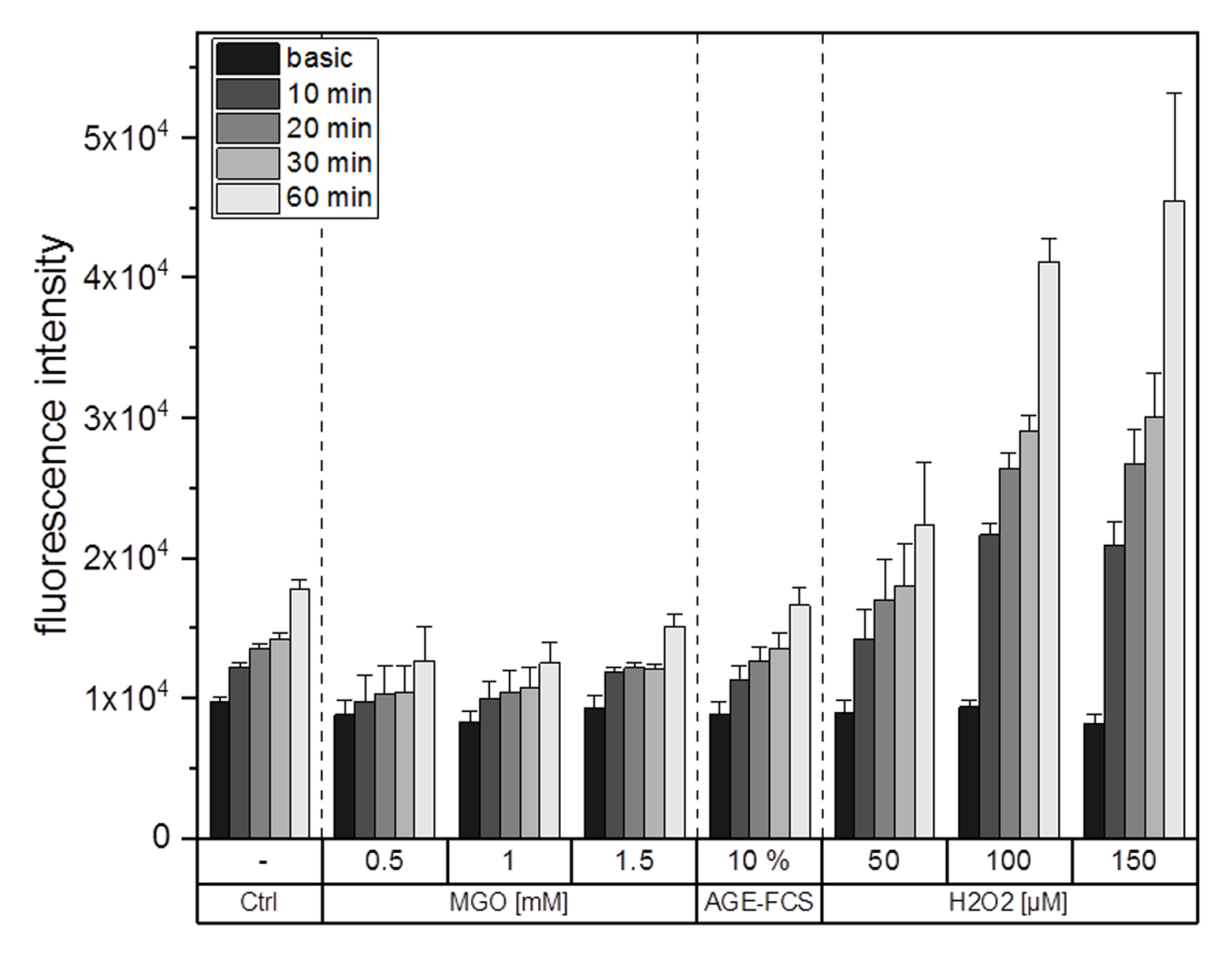
**ROS production after glycation.** THP-1 macrophages (M0) were treated with different MGO concentrations, 10% AGE-FCS or different concentrations of H_2_O_2_ for up to 60 min. Production of intracellular ROS was determined using fluorescent probe H_2_DCFDA and measurement of fluorescence intensity. Basic measurement represents fluorescence intensity after loading of the cells with H_2_DCFDA but without addition of any treatment. Shown is one representative graph of 3 independent measurements. Data represents average mean + SD of 5 technical replicates.

It is well known that glycation and AGE-signaling can increase cellular ROS levels. To clarify whether glycation using MGO or the treatment with AGE-FCS induce production of cellular ROS, we performed ROS measurements using an H_2_DCFDA assay. Figure 6 shows one representative graph for ROS measurements. The values which are entitled “basic” represent the fluorescence signal of the samples after loading with H_2_DCFDA but without any treatments. Increasing concentrations of H_2_O_2_ were used as positive controls for ROS induction. We could show that treatment with 0.5, 1 or 1.5 mM MGO as well as 10% AGE-FCS did not increase ROS production compared to the control. However, treatment with H_2_O_2_ did raise cellular ROS levels in a time and concentration dependent manner.

### Glycation increases cytokine expression

**Figure 7:**
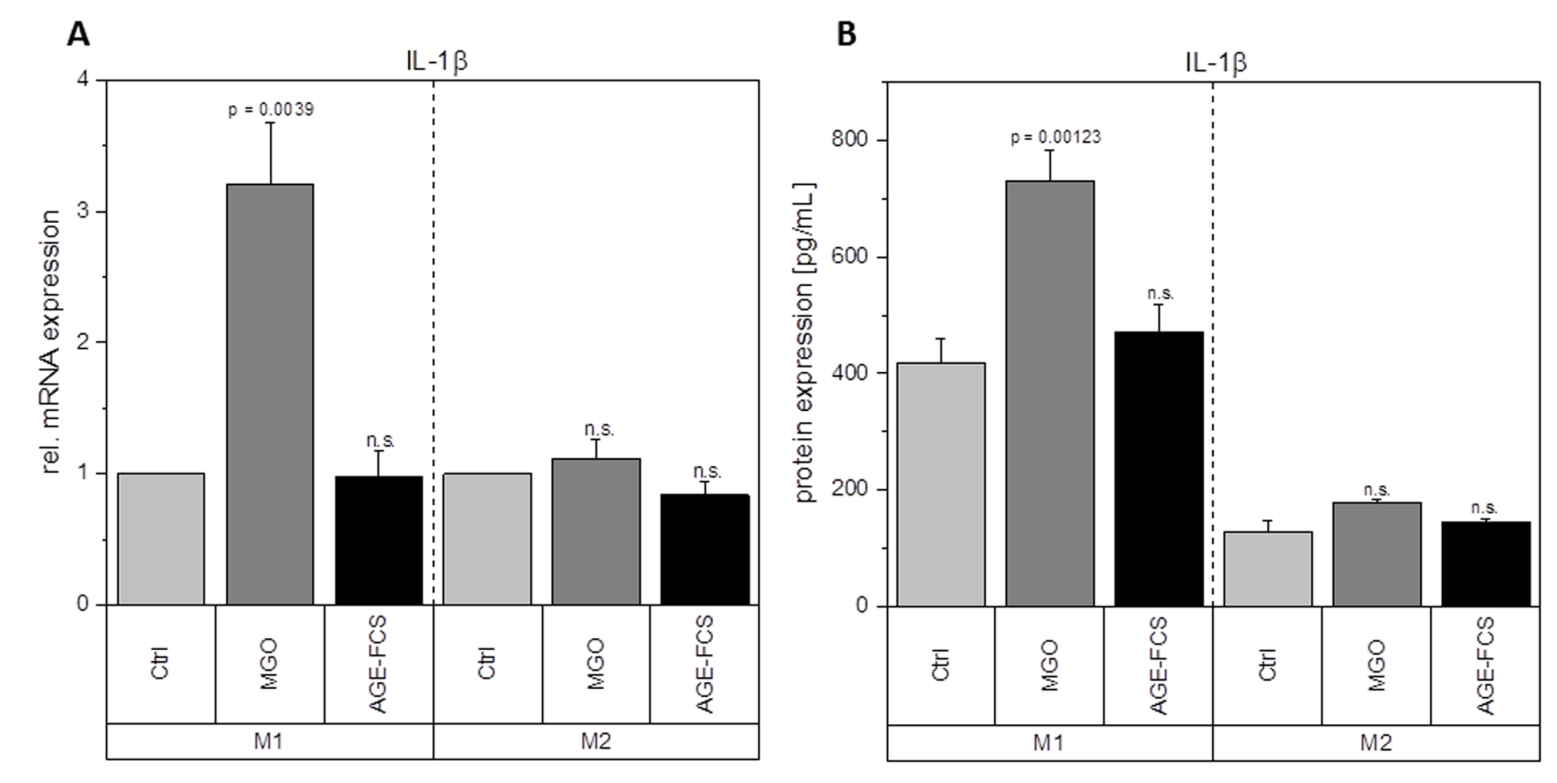
**Expression of IL-1β after glycation.** THP-1 macrophages were glycated with 1 mM MGO or treated with 10% AGE-FCS and polarized in M1 or M2 phenotype. Expression of IL-1β was quantified using qPCR (**A**). Data was normalized to untreated control cells. Graph shows average mean of relative mRNA expression + SD of 3 independent experiments. Protein secretion of IL-1β was quantified in the cell supernatant using cytometric bead array (**B**). Graph shows average mean of IL-1β concentration (in pg/mL) + SD of 3 independent experiments.

**Figure 8:**
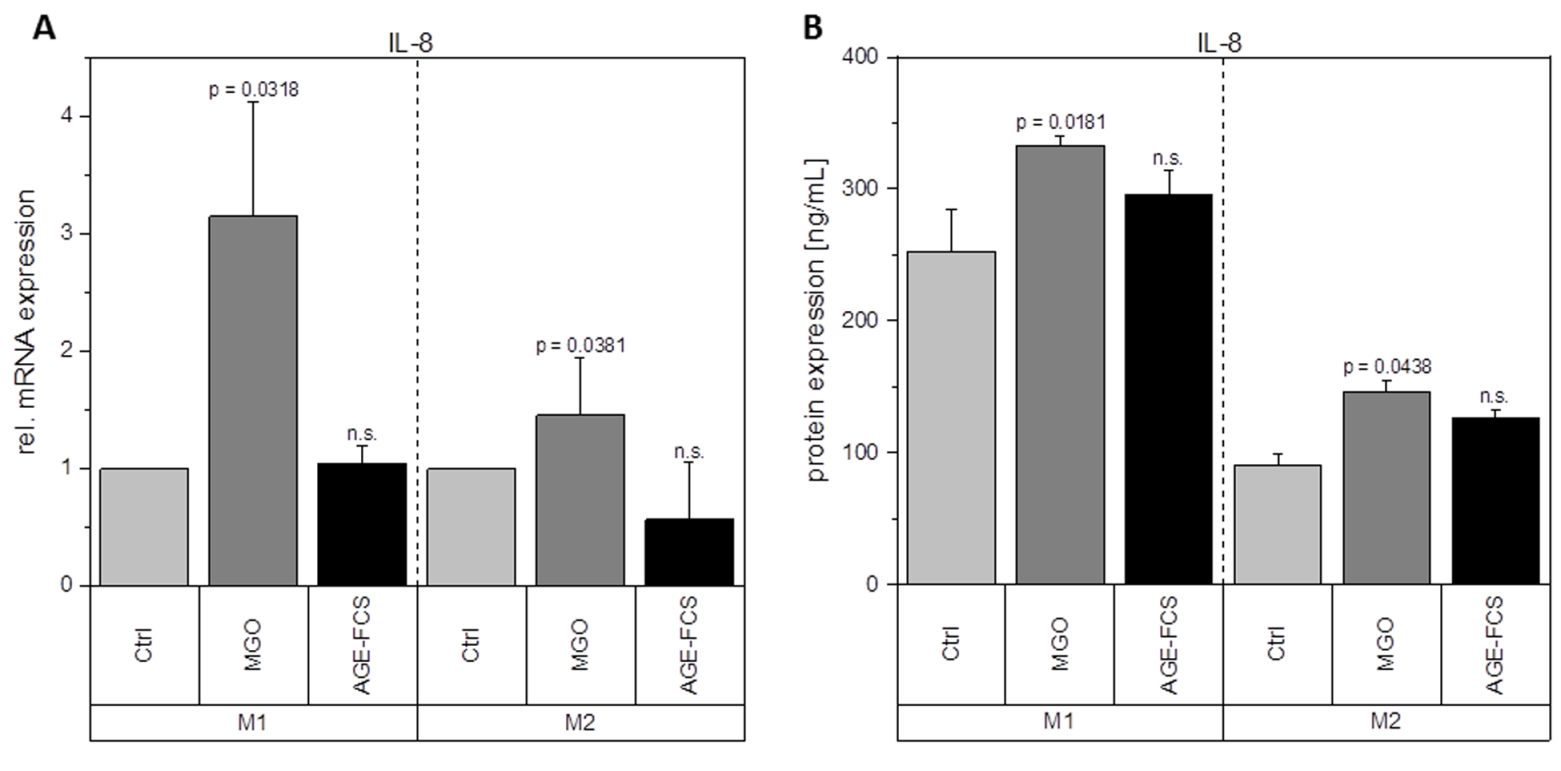
**Expression of IL-8 after glycation.** THP-1 macrophages were glycated with 1 mM MGO or treated with 10% AGE-FCS and polarized in M1 or M2 phenotype. Expression of IL-8 was quantified using qPCR (**A**). Data was normalized to untreated control cells. Graph shows average mean of relative mRNA expression + SD of 3 independent experiments. Protein secretion of IL-8 was quantified in the cell supernatant using cytometric bead array (**B**). Graph shows average mean of IL-8 concentration (in ng/mL) + SD of 3 independent experiments.

**Figure 9:**
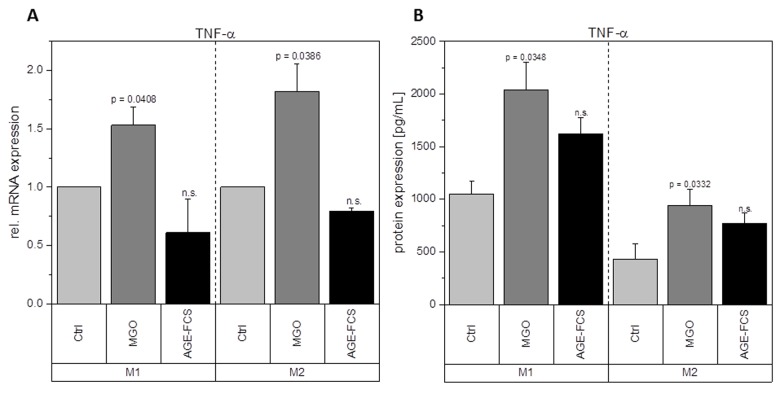
**Expression of TNF-α after glycation.** THP-1 macrophages were glycated with 1 mM MGO or treated with 10% AGE-FCS and polarized in M1 or M2 phenotype. Expression of TNF-α was quantified using qPCR (**A**). Data was normalized to untreated control cells. Graph shows average mean of relative mRNA expression + SD of 3 independent experiments. Protein secretion of TNF-α was quantified in the cell supernatant using cytometric bead array (**B**). Graph shows average mean of TNF-α concentration (in pg/mL) + SD of 3 independent experiments.

**Figure 10:**
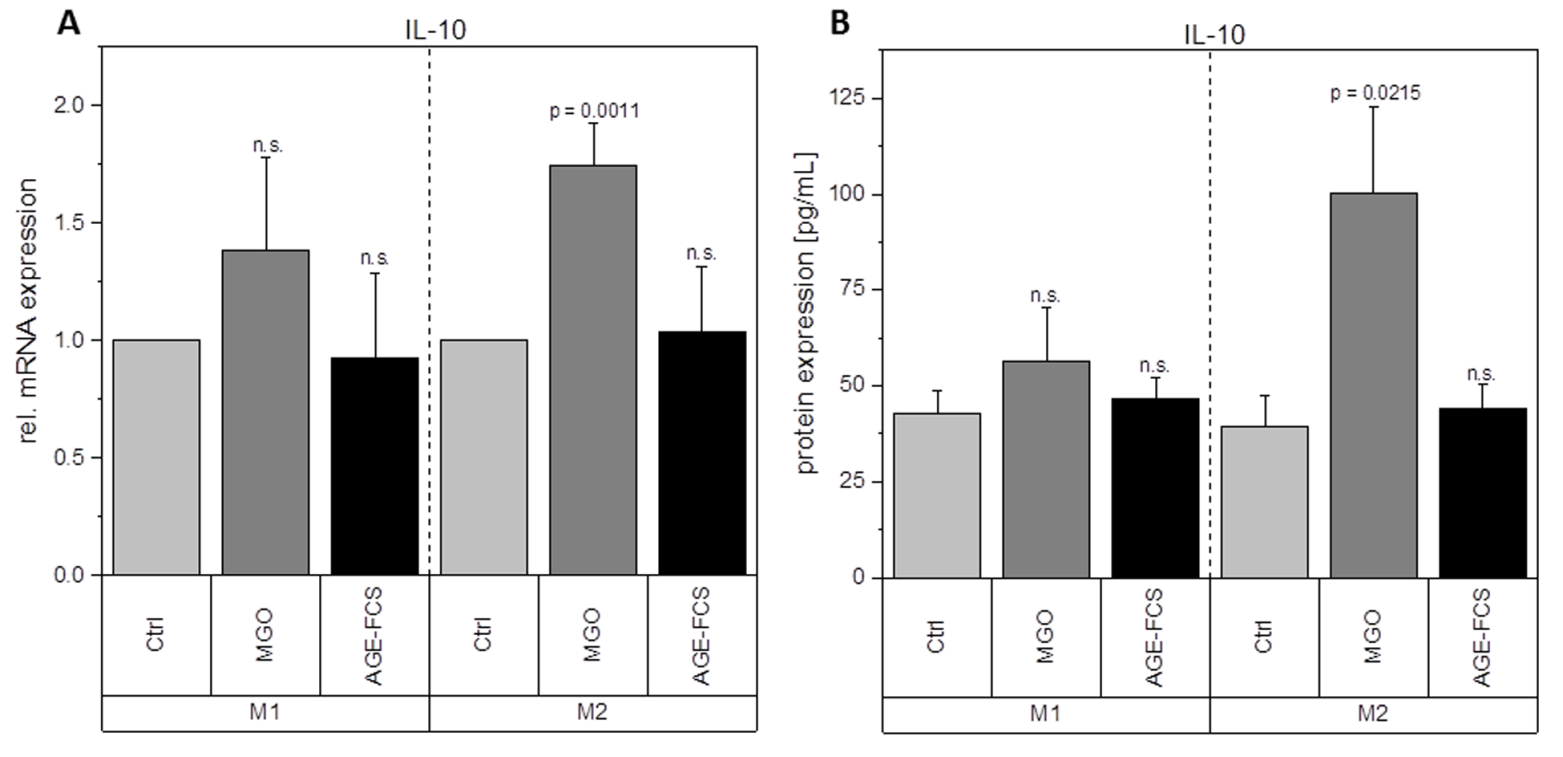
**Expression of IL-10 after glycation.** THP-1 macrophages were glycated with 1 mM MGO or treated with 10% AGE-FCS and polarized in M1 or M2 phenotype. Expression of IL-10 was quantified using qPCR (**A**). Data was normalized to untreated control cells. Graph shows average mean of relative mRNA expression + SD of 3 independent experiments. Protein secretion of IL-10 was quantified in the cell supernatant using cytometric bead array (**B**). Graph shows average mean of IL-10 concentration (in pg/mL) + SD of 3 independent experiments.

Macrophages are key players during inflammation. Therefore, we analyzed expression of inflammation-related cytokines in M1 and M2 macrophages after glycation with 1 mM MGO or treatment with 10% AGE-FCS. Figure 7A shows relative mRNA levels for pro-inflammatory cytokine IL-1β. M1 macrophages showed a significant increase of IL-1β mRNA after treatment with MGO, however, there was no change after treatment with AGE-FCS. For M2 macrophages we could also not detect any changes in mRNA levels, both for MGO and AGE-FCS treatment. We could also confirm these findings on protein levels (Figure 7B) in the supernatant. Due to this increase of IL-1β in M1 macrophages, we wanted to elucidate whether the inflammasome is involved. We checked for caspase-1 expression using immunoblotting after 4, 8 and 24 h in M1 and M2 macrophages treated with MGO or AGE-FCS. However, we could not detect any upregulation of caspase-1 compared to the untreated controls in any of the treatments. Regarding expression of pro-inflammatory cytokine IL-8, we could show an increase of mRNA expression levels (Figure 8A) after treatment with MGO for both M1 and M2 macrophages, while treatment with AGE-FCS did not have any effect. We could also confirm these findings on protein levels (Figure 8B) in the supernatant. We also analyzed TNF-α as a multifactorial cytokine involved in inflammation and could show an increase of mRNA expression levels for both M1 and M2 macrophages treated with MGO (Figure 9A). Treatment with AGE-FCS did not lead to any changes on expression of TNF-α. These findings could be confirmed on protein levels (Figure 9B). Regarding anti-inflammatory cytokine IL-10, we measured an increase in mRNA levels (Figure 10A) of M2 macrophages treated with MGO, while all other samples remained unaffected. Again, this increase could be confirmed on protein levels (Figure 10B).

### Phagocytic efficiency is reduced after glycation

**Figure 11:**
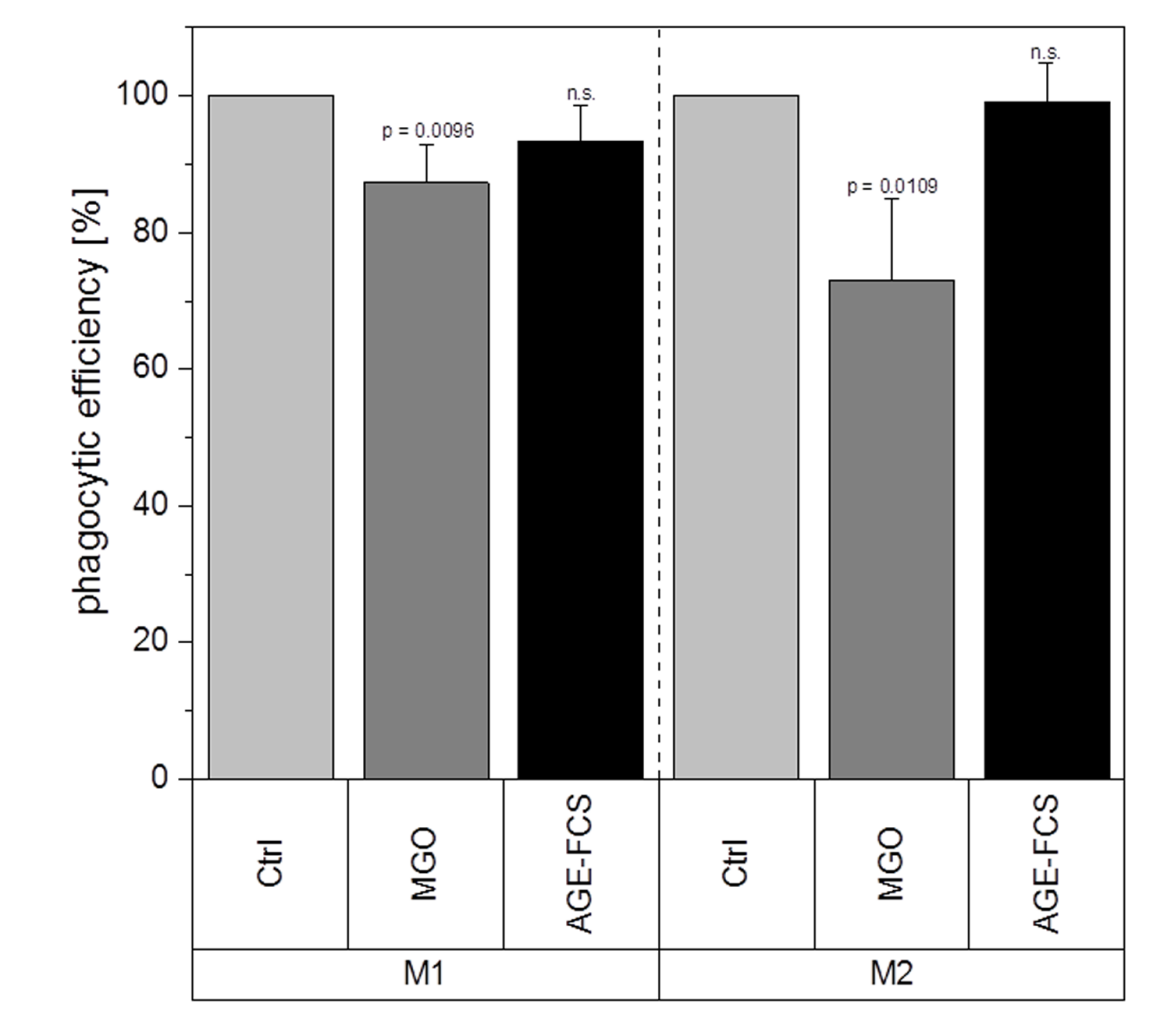
**Phagocytic efficiency after glycation.** THP-1 macrophages were glycated with 1 mM MGO or treated with 10% AGE-FCS for 24 h and polarized in M1 or M2 phenotype. Phagocytosis assay was performed with pHrodo™ Green *E. coli* BioParticles™. Data was normalized to untreated control cells. Graphs show average mean of phagocytic efficiency + SD of 5 independent experiments.

We further wanted to investigate whether MGO treatment has any effect on macrophage function. Therefore, we analyzed their phagocytic efficiency after treatment with 1 mM MGO or 10% AGE-FCS. Phagocytosis assay with pHrodo™ Green *E. coli* BioParticles™ was performed and change of phagocytic efficiency was calculated and compared to untreated cells. Macrophages treated with MGO showed a significant decrease of phagocytic efficiency (Figure 11). We could observe a reduction of 13 ± 5% (mean ± SD; p = 0.0096) for the M1 phenotype and 27 ± 12% (mean ± SD; p = 0.0109) for the M2 phenotype. In comparison, treatment with AGE-FCS did not have any significant effects.

## DISCUSSION

Glycation and accumulation of AGEs are known to have negative effects on protein function and maintenance of cellular homeostasis. During the process of ageing, there is a decline in immune functions and immune responses, resulting in health implications and immune deficiency [42,43]. Although it is known that AGEs are responsible for several age-related diseases, there is still not much known about their impact on the immune system in general. In this study, we could show that MGO treatment led to an increase in cellular formation of AGEs in macrophages and the level of AGE correlated with the MGO concentrations used. MGO concentrations up to 1 mM did not lead to significant decreases in metabolic activity of macrophages. Reduced metabolic activity and induction of apoptosis could only be detected after incubation with 1.5 or 2 mM MGO. In other cell lines, e.g. murine alveolar macrophages, even lower concentrations of MGO (0.4 and 0.8 mM) were able to induce apoptosis and necrosis [38]. The used MGO concentrations in this study also did not induce cellular production of ROS in macrophages. Contrary, in peritoneal macrophages of tumor mice an induction of superoxide anion and nitrite production as well as ROS levels could be observed after MGO treatment [39]. In general, phagocytes can produce high levels of ROS and reactive carbonyl species upon exposure to MGO, leading to inflammation, apoptosis and necrosis, as well as release of several cytokines [62]. Our data indicates that the effects we observed are not only based on higher ROS levels and oxygen stress.

Glycation upregulates mRNA expression of pro-inflammatory cytokine IL-1β in M1 macrophages and TNF-α and IL-8 in both M1 and M2 macrophages. It also enhances the mRNA and protein expression levels of anti-inflammatory cytokine IL-10 in M2 macrophages. This could also be verified on protein levels. Although treatment with AGE-FCS increases the expression of RAGE, there was no effect on these mRNA or protein expression levels. We aimed to exclude that our findings are influenced by glycated serum proteins in the cell culture medium. Therefore, we always used medium containing 10% AGE-FCS as a control, that was glycated under the same conditions as we glycated the cells, in order to verify that our results are linked to direct glycation of the macrophages.

IL-1β is produced by activated monocytes and macrophages. It is secreted during infections, inflammatory processes, or microbial invasion, and functions in both systemic and local response mechanisms [44]. Increased concentrations of IL-1β could be found in diabetic wounds, which can be correlated with a positive feedback loop that sustains the pro-inflammatory macrophage phenotype observed in poorly healing wounds [37]. It was also suggested that IL-1β blocks the induction of M2 phenotype, which can be observed during normal healing processes [37]. Our findings show that glycation of cellular proteins but not AGE-FCS, induce an overexpression of IL-1β in M1 macrophages and therefore corroborate these studies. Besides, other studies also showed increased IL-1β expression in murine peritoneal macrophages of tumor mice after treatment with MGO [39,40]. Regarding increased IL-1β concentrations, it can be beneficial for further understanding to investigate if the inflammasome is involved and maybe also stronger activated. We focused on caspase-1 expression in order to determine inflammasome activity [45,46]. We could not detect any changes of caspase-1 expression upon treatment with MGO or AGE-FCS. We therefore estimate that the inflammasome is not additionally activated by glycation. IL-8 has extensive functions in defensive and immune reactions as well as in inflammation [47,48]. During acute inflammation, IL-8 is highly expressed in M1 macrophages and turned off during resolution [49,50]. Increased IL-8 expression in M1 macrophages due to glycation indicates a prolonged inflammation phase. Our data also show an upregulation in M2 macrophages, which should only produce low levels of IL-8, indicating that glycation also triggers anti-inflammatory macrophages to a more pro-inflammatory phenotype. TNF-α is involved in inflammation as a pleiotropic cytokine and can be seen as a master regulator for the production and secretion of pro-inflammatory cytokines [51]. We could show that glycation increases secretion of TNF-α in both M1 and M2 macrophages. For M1 macrophages this contributes to the pro-inflammatory activation of glycation we already observed. For M2 macrophages it indicates a severe change in their anti-inflammatory phenotype. Secretion of TNF-α during remodeling phase of wound healing can promote tissue damage and apoptosis.

IL-10 as one of the most potent anti-inflammatory cytokines is able to inhibit pro-inflammatory cytokine production. IL-10 also restrains immune response and interferes with immune cell functions, including those of macrophages [52,53]. Our data show no significant effects of glycation on IL-10 expression in M1 macrophages. This supports our assumption that AGEs and glycation increase pro-inflammatory signaling. IL-10 production is known to reduce M1 macrophage activation and cytotoxicity and also increases M2 macrophage activation [54]. If IL-10 is maintained at a basal level, the overexpression of pro-inflammatory cytokines like IL-1β, IL-8 and TNF-α could mask IL-10 effects on the polarization-switch to an anti-inflammatory phenotype. Regarding M2 phenotype, we could demonstrate that IL-10 expression is upregulated after glycation. This effect could be due to the upregulation of IL-8 and TNF-α, which possibly triggers M2 macrophages to shift to a more pro-inflammatory phenotype. IL-10 upregulation could therefore indicate a self-regulating reaction of the cell to stay in its anti-inflammatory phenotype.

Even though our data showed increased expression of pro-inflammatory cytokines, we could detect a significant decline in phagocytic efficiency for both M1 and M2 macrophages after glycation. We believe that this dysfunction is due to glycation of cell surface proteins as shown before (Figure 2B), influencing the binding efficiency of phagocytosis relevant receptors like toll-like receptors [55,56]. Phagocytosis of invading microbes, but also of cell debris or apoptotic cells, is important during acute infections as well as during tissue remodeling [57]. Reduced phagocytosis due to glycation could be supportive for prolonged inflammation and also contribute to chronic wounds in diabetic patients.

Taken all this together, our data indicate for the first time that glycation and cellular AGE formation indeed affect activation of human macrophages. It has been shown by other groups that treatment with AGE-modified proteins led to an upregulation of RAGE expression [43,44]. We could also see an upregulation of RAGE after treatment with AGE-FCS, but not after direct glycation of the cells using MGO. We could exclude that the effects we observed result from external AGE binding through RAGE activation. It is well known that activation of RAGE and its signaling cascade can upregulate the production of pro-inflammatory cytokines and chemokines in monocytes and macrophages, for instance such as IL-1β [58]. However, we could not show any upregulated expression of pro-inflammatory cytokines after treatment with AGE-FCS, even though we could show upregulation of RAGE, indicating that our findings result from glycation of the macrophages and not from RAGE activation. This could be a hint for the underlying mechanisms how glycation influences cell behavior. However, we believe that the impact of glycation on macrophage activation is much more complex and still needs to be further investigated.

## MATERIALS AND METHODS

### Reagents and cells

Methylglyoxal (MGO), dimethyl sulfoxide (DMSO), hydrogen peroxide solution (H_2_O_2_) and 3-(4,5-Dimethylthiazol-2-yl)-2,5-diphenyltetrazolium bromide (MTT) were purchased from Sigma-Aldrich (St. Louis, USA). 12-Myristate 13-acetate (PMA; Sigma-Aldrich) was diluted in DMSO to a final concentration of 0.1 mg/mL and aliquoted after sterile filtration. β-mercaptoethanol (β-ME) was used from Thermo Fisher Scientific Inc. (Waltham, USA). Lipopolysaccharide (LPS) from *Escherichia coli* (strain O111:B4) was obtained from Sigma-Aldrich and diluted in PBS to a final concentration of 1 µg/mL. Human recombinant interferon-γ (IFN-γ), IL-4 & IL-13 were purchased from ImmunoTools (Friesoythe, Germany) and diluted to 1 µg/mL in deionized water. APC Annexin V Apoptosis Detection Kit with 7-aminoactinomycin (7AAD) was purchased from BioLegend (San Diego, USA). 2',7'-dichlorodihydrofluorescein diacetate (H_2_DCFDA) was purchased from Thermo Fisher Scientific Inc.

The human monocytic cell line THP-1 was a kind gift from Dr. Jörg Lehmann (Fraunhofer Institute for Cell Therapy and Immunology, Leipzig, Germany). Cultivation was done in RPMI 1640 (with L-glutamine; Lonza Group Ltd., Basel, Switzerland) supplemented with 10% fetal calf serum (FCS; GE Helathcare, Little Chalfont, UK), 100 units/mL penicillin and 100 µg/mL streptomycin (Thermo Fisher Scientific Inc.) at 37 °C and 5% CO_2_ in a humidified incubator.

### Preparation of glycated FCS

FCS with or without addition of 1 mM MGO was incubated at 37 °C for 24 h. FCS was stored at -20 °C until use. Glycated FCS is further stated to as AGE-FCS. Glycation of AGE-FCS was confirmed via dot blot with anti-AGE antibody CML-26 (Abcam, Cambridge, UK) at a concentration of 0.05 µg/mL. We could demonstrate that our AGE-FCS is strongly glycated and the untreated FCS is not (Supplementary Figure 4).

### Differentiation and stimulation of cells

THP-1 cells (2 x 10^6^ cells, if not stated otherwise) were differentiated into macrophages using 100 ng/mL PMA & 50 µM β-ME diluted in normal growth medium (further stated as differentiation medium) for 48 h. Differentiated macrophages (M0) can be further polarized via incubation for 24 h with 100 ng/mL LPS and 20 ng/mL rh IFN-γ for M1 phenotype or with 20 ng/mL rh IL-4 and rh IL-13 for M2 phenotype (according to [59–61]) (Figure 1).

If not stated otherwise, cells were treated with 1 mM MGO in normal growth medium or with medium containing 10% AGE-FCS for 24 h, untreated cells grown in normal growth medium served as controls. Medium was removed from the cells via aspiration and macrophages were polarized either into M1 or into M2 phenotype as stated before. Treated macrophages were harvested after incubation with Accutase® (BioLegend) for up to 30 min and pelleted by centrifugation (160 g, 3 min).

### Metabolic activity

Metabolic activity of glycated THP-1 cells was measured using an MTT assay. Macrophages were seeded into 96-well microtiter plates at a density of 5 x 10^4^ cells per well. After treatment, cells were washed with 200 µL PBS per well. MTT was diluted to a final concentration of 0,5 mg/mL in normal growth medium and cells were incubated for 4 h with 100 µL MTT solution per well. After removal of the MTT containing medium, remaining formazan crystals were dissolved in 150 µL DMSO. Absorption values were measured at a wavelength of 570 nm (background 630 nm). Untreated control cells were then set to 100% of metabolic activity and changes in metabolic activity of treated cells were calculated.

### Apoptosis assay

Apoptosis assay was performed as described previously [41]. The percentage of Annexin V^-^ / 7AAD^-^ cells was used to determine the number of intact living cells (= non-apoptotic and non-necrotic).

### ROS measurement

Changes in the production of intracellular ROS can be demonstrated using the fluorescent probe H_2_DCFDA. For ROS measurement, macrophages were used at a density of 1 x 10^5^ cells per well in 96-well microtiter plates. Cells were loaded with 100 µL H_2_DCFDA (diluted to 10 µM in PBS) per well and incubated for 10 min. H_2_DCFDA was removed and replaced by 100 µL normal growth medium. Basic fluorescence intensity was measured in a plate reader at 495 nm excitation and 525 nm emission. Medium was removed and treatments were applied (100 µL / well, n = 5). Different concentrations of H_2_O_2_ were used as positive controls for ROS induction. Fluorescence intensity was measured as mentioned above after 10, 20, 30 and 60 min of incubation.

### Immunofluorescence staining

For immunofluorescence (IF) staining, 5 x 10^4^ cells were directly seeded in differentiation medium into 8-well chamber slides. After treatment, cells were washed with 200 µL PBS and fixed with 4% paraformaldehyde for 15 min, washed again 3 times with 0.1% Tween®20 in PBS and blocked for 15 min with 0.3% FCS in PBS. After 3 washing steps with 200 µL of 0.3% FCS, cells were stained for 1 h with CML-26 antibody (0.5 µg/mL in 0.3% FCS). The previous washing step was repeated, followed by staining with secondary fluorescein-coupled antibody (20 µg/mL; Thermo Fisher Scientific Inc.) and Hoechst staining (5 µg/mL, Sigma-Aldrich) for 30 min. Cells were washed 3 times with 200 µL of 0.1% Tween®20 and coverslips were applied using ClearMount™ solution (Thermo Fisher Scientific Inc.). Images were taken using an Axio Observer 7 microscope (Zeiss, Jena, Germany) with a 20x objective.

### Immunoblotting

Total protein was either isolated from cell pellets after 24 h of incubation or cells were directly lysed in hot SDS-sample buffer after 4 h of incubation. Samples were separated via SDS-PAGE (10% or 12%) and transferred to a nitrocellulose membrane using western blot techniques. Glycation was detected via anti-AGE antibody CML-26 (0.05 µg/mL), RAGE was detected via anti-RAGE antibody ab3611 (1 µg/mL; Abcam) and Caspase-1 was detected via anti-Caspase-1 antibody (0.182 µg/mL; Cell Signaling Technology, Cambridge, UK). Secondary peroxidase-coupled antibody (Immuno Research Inc., Eagan, USA) was detected by enhanced chemiluminescence. Images were taken using Chemidoc XRS imaging system (Bio-Rad Laboratories, Hercules, USA). Ponceau S staining of total loaded protein and second staining with anti-actin antibody Ab-5 (0.05 µg/mL; BD Biosciences, Franklin Lakes, USA) was used as loading control. For quantification, band intensity of proteins of interest was transformed into numeric values using Image J (Wayne Rasband, National Institutes of Health, Bethesda, USA) and normalized to the corresponding loading controls.

### Quantitative real-time PCR

Total RNA was isolated from cell pellets by column-based method (Quick-RNA™ MiniPrep Kit, Zymo Research, Irvine, USA) according to manufacturer’s instructions including DNAse I-treatment. Concentration and quality of isolated RNA was spectrophotometrically assessed (NanoDrop, Thermo Fisher Scientific Inc.). For reverse transcription 2 µg RNA was used (SuperScript™ II Reverse Transcriptase, Thermo Fisher Scientific Inc.) and proceeded according to manufacturer’s instructions. Quantitative real-time PCR (qPCR) was performed using iQ™5 Multicolor Real-Time PCR Detection System (Bio-Rad Laboratories Inc., Hercules, California, USA) and qPCR GreenMaster (Jena Bioscience, Jena, Germany). Primer sets for IL-1β, IL-8, IL-10 and TNF-α were used according to [62]. Ribosomal protein L32 (RPL32) according to [63] was used as housekeeping gene to normalize data. All reactions were done in triplicates. ΔΔCt method was used for data analysis. Values of genes of interest were first subtracted from the values of RPL32 (ΔCt). N-fold change of gene expression was then calculated as 2^-(ΔCt treated – ΔCt untreated)^.

### Cytokine quantification

Cell supernatants were collected 24 h post polarization and cytokine quantification was performed by cytometric bead array CBA Flex (Human Soluble Protein Master Buffer Kit, BD Biosciences) detecting simultaneously IL-1β (Human IL-1β Flex Set), IL-8 (Human IL-8 Flex Set), IL-10 (Human IL-10 Flex Set) and TNF-α (Human TNF Flex Set) according to the manufacturer’s recommendation. For the detection of IL-8, samples were diluted 1:500; all other samples were not diluted. Samples were analyzed with the flow cytometer FACSVerse™ (BD Biosciences). Cytokine concentrations were calculated according to internal standard curves. Final analysis and calculation was carried out using FCAP Array™ software (BD Biosciences).

### Phagocytosis assay

For analysis of the phagocytic efficiency, macrophages were used at a density of 1 x 10^5^ cells per well in 96-well-plates. Macrophages were washed twice with 200 µL PBS after treatment and incubated with 100 µL pHrodo™ Green *E. coli* BioParticles™ (60 µg/mL; Thermo Fisher Scientific Inc.) solution for 1 h. This special dye is non-fluorescent outside the cell at neutral pH, but fluoresces brightly green at acidic pH, such as in phagosomes. After removal of the *E. coli* BioParticles, cells were incubated with 150 µL Accutase® for 30 min and harvested. Five wells per sample were united and centrifuged. Cell pellets were then re-suspended in 200 µL Live Cell Imaging Solution (LCIS, Thermo Fisher Scientific Inc.). Analysis of 10,000 cells per sample was done using BD Accuri™ C6 flow cytometer (BD Biosciences). Non-glycated cells without *E.coli* addition (incubated in LCIS) were used for gating. Phagocytosis rate of non-glycated control cells was set to 100% and percentage change of phagocytosis was calculated for treated cells.

### Flow cytometry staining

For flow cytometry staining, THP-1 macrophages (M0) were treated with 1 mM MGO or 10% AGE-FCS for 24 h and harvested using 0.25% PBS-EDTA. Cells were washed with PBS and incubated for 1 h at 4 °C with anti-RAGE antibody ab3611 (40 µg/mL in LCIS). After a washing step with PBS, cells were incubated with secondary FITC-labeled antibody (10 µg/mL; Thermo Fisher Scientific Inc.) for 30 min at 4 °C. After another washing step with PBS, cells were re-suspended in 200 µL LCIS. Analysis of 10,000 cells per sample was done with BD Accuri™ C6 flow cytometer (BD Biosciences) using the FL-1 channel (excitation 488 nm, 530 / 30 nm band pass filter).

### Statistical analysis

All analyses and visualizations were performed using OriginPro 2018 software (OriginLab Corporation, Northampton, USA). Paired student t-test against the control group or a theoretical value of 1 (due to data normalization) was used. Figures show the average mean + standard deviation (SD) and levels of significance are represented within the figures.

## Supplementary Material

Supplementary Figures

## References

[1] Wu JT. Advanced glycosylation end products: a new disease marker for diabetes and aging. J Clin Lab Anal. 1993; 7:252–55. 10.1002/jcla.1860070503 8410484

[2] Yamagishi S. Role of advanced glycation end products (AGEs) and receptor for AGEs (RAGE) in vascular damage in diabetes. Exp Gerontol. 2011; 46:217–24. 10.1016/j.exger.2010.11.007 21111800

[3] Jakus V, Rietbrock N. Advanced glycation end-products and the progress of diabetic vascular complications. Physiol Res. 2004; 53:131–42. 15046548

[4] Angeloni C, Zambonin L, Hrelia S. Role of methylglyoxal in Alzheimer’s disease. BioMed Res Int. 2014; 2014:238485. 10.1155/2014/238485 24734229 PMC3966409

[5] Drenth H, Zuidema SU, Krijnen WP, Bautmans I, van der Schans C, Hobbelen H. Association between advanced glycation end-products and functional performance in Alzheimer’s disease and mixed dementia. Int Psychogeriatr. 2017; 29:1525–34. 10.1017/S1041610217000886 28539135

[6] Wetzels S, Wouters K, Schalkwijk CG, Vanmierlo T, Hendriks JJ, Kleinschnitz C. Methylglyoxal-Derived Advanced Glycation Endproducts in Multiple Sclerosis. Int J Mol Sci. 2017; 18:E421. 10.3390/ijms18020421 28212304 PMC5343955

[7] López-Díez R, Shekhtman A, Ramasamy R, Schmidt AM. Cellular mechanisms and consequences of glycation in atherosclerosis and obesity. Biochim Biophys Acta. 2016; 1862:2244–52. 10.1016/j.bbadis.2016.05.005 27166197 PMC5101176

[8] Vistoli G, De Maddis D, Cipak A, Zarkovic N, Carini M, Aldini G. Advanced glycoxidation and lipoxidation end products (AGEs and ALEs): an overview of their mechanisms of formation. Free Radic Res. 2013 (Suppl 1); 47:3–27. 10.3109/10715762.2013.815348 23767955

[9] Thornalley PJ. Dicarbonyl intermediates in the maillard reaction. Ann N Y Acad Sci. 2005; 1043:111–17. 10.1196/annals.1333.014 16037229

[10] Phillips SA, Thornalley PJ. The formation of methylglyoxal from triose phosphates. Investigation using a specific assay for methylglyoxal. Eur J Biochem. 1993; 212:101–05. 10.1111/j.1432-1033.1993.tb17638.x 8444148

[11] Ahmed N, Battah S, Karachalias N, Babaei-Jadidi R, Horányi M, Baróti K, Hollan S, Thornalley PJ. Increased formation of methylglyoxal and protein glycation, oxidation and nitrosation in triosephosphate isomerase deficiency. Biochim Biophys Acta. 2003; 1639:121–32. 10.1016/j.bbadis.2003.08.002 14559119

[12] Rabbani N, Thornalley PJ. Dicarbonyl stress in cell and tissue dysfunction contributing to ageing and disease. Biochem Biophys Res Commun. 2015; 458:221–26. 10.1016/j.bbrc.2015.01.140 25666945

[13] Schmidt AM, Yan SD, Yan SF, Stern DM. The multiligand receptor RAGE as a progression factor amplifying immune and inflammatory responses. J Clin Invest. 2001; 108:949–55. 10.1172/JCI200114002 11581294 PMC200958

[14] Neeper M, Schmidt AM, Brett J, Yan SD, Wang F, Pan YC, Elliston K, Stern D, Shaw A. Cloning and expression of a cell surface receptor for advanced glycosylation end products of proteins. J Biol Chem. 1992; 267:14998–5004. 1378843

[15] Sevillano N, Girón MD, Salido M, Vargas AM, Vilches J, Salto R. Internalization of the receptor for advanced glycation end products (RAGE) is required to mediate intracellular responses. J Biochem. 2009; 145:21–30. 10.1093/jb/mvn137 18849572

[16] Yan SD, Schmidt AM, Anderson GM, Zhang J, Brett J, Zou YS, Pinsky D, Stern D. Enhanced cellular oxidant stress by the interaction of advanced glycation end products with their receptors/binding proteins. J Biol Chem. 1994; 269:9889–97. 8144582

[17] Ramasamy R, Yan SF, Schmidt AM. The diverse ligand repertoire of the receptor for advanced glycation endproducts and pathways to the complications of diabetes. Vascul Pharmacol. 2012; 57:160–67. 10.1016/j.vph.2012.06.004 22750165 PMC3433629

[18] Kilhovd BK, Juutilainen A, Lehto S, Rönnemaa T, Torjesen PA, Birkeland KI, Berg TJ, Hanssen KF, Laakso M. High serum levels of advanced glycation end products predict increased coronary heart disease mortality in nondiabetic women but not in nondiabetic men: a population-based 18-year follow-up study. Arterioscler Thromb Vasc Biol. 2005; 25:815–20. 10.1161/01.ATV.0000158380.44231.fe 15692098

[19] Mallipattu SK, Uribarri J. Advanced glycation end product accumulation: a new enemy to target in chronic kidney disease? Curr Opin Nephrol Hypertens. 2014; 23:547–54. 10.1097/MNH.0000000000000062 25160075 PMC5577004

[20] Strain WD, Hope SV, Green A, Kar P, Valabhji J, Sinclair AJ. Type 2 diabetes mellitus in older people: a brief statement of key principles of modern day management including the assessment of frailty. A national collaborative stakeholder initiative. Diabet Med. 2018; 35:838–45. 10.1111/dme.13644 29633351

[21] Whiting DR, Guariguata L, Weil C, Shaw J. IDF diabetes atlas: global estimates of the prevalence of diabetes for 2011 and 2030. Diabetes Res Clin Pract. 2011; 94:311–21. 10.1016/j.diabres.2011.10.029 22079683

[22] McLellan AC, Thornalley PJ, Benn J, Sonksen PH. Glyoxalase system in clinical diabetes mellitus and correlation with diabetic complications. Clin Sci (Lond). 1994; 87:21–29. 10.1042/cs0870021 8062515

[23] Bierhaus A, Hofmann MA, Ziegler R, Nawroth PP. AGEs and their interaction with AGE-receptors in vascular disease and diabetes mellitus. I. The AGE concept. Cardiovasc Res. 1998; 37:586–600. 10.1016/S0008-6363(97)00233-2 9659442

[24] Basu Mallik S, Jayashree BS, Shenoy RR. Epigenetic modulation of macrophage polarization- perspectives in diabetic wounds. J Diabetes Complications. 2018; 32:524–30. 10.1016/j.jdiacomp.2018.01.015 29530315

[25] Singh N, Armstrong DG, Lipsky BA. Preventing foot ulcers in patients with diabetes. JAMA. 2005; 293:217–28. 10.1001/jama.293.2.217 15644549

[26] Davis FM, Kimball A, Boniakowski A, Gallagher K. Dysfunctional Wound Healing in Diabetic Foot Ulcers: new Crossroads. Curr Diab Rep. 2018; 18:2. 10.1007/s11892-018-0970-z 29362914

[27] Mulder GD, Patt LM, Sanders L, Rosenstock J, Altman MI, Hanley ME, Duncan GW. Enhanced healing of ulcers in patients with diabetes by topical treatment with glycyl-l-histidyl-l-lysine copper. Wound Repair Regen. 1994; 2:259–69. 10.1046/j.1524-475X.1994.20406.x 17147644

[28] Brem H, Tomic-Canic M. Cellular and molecular basis of wound healing in diabetes. J Clin Invest. 2007; 117:1219–22. 10.1172/JCI32169 17476353 PMC1857239

[29] Falanga V. Wound healing and its impairment in the diabetic foot. Lancet. 2005; 366:1736–43. 10.1016/S0140-6736(05)67700-8 16291068

[30] Baltzis D, Eleftheriadou I, Veves A. Pathogenesis and treatment of impaired wound healing in diabetes mellitus: new insights. Adv Ther. 2014; 31:817–36. 10.1007/s12325-014-0140-x 25069580

[31] Mantovani A, Sica A, Locati M. Macrophage polarization comes of age. Immunity. 2005; 23:344–46. 10.1016/j.immuni.2005.10.001 16226499

[32] Martinez FO, Helming L, Gordon S. Alternative activation of macrophages: an immunologic functional perspective. Annu Rev Immunol. 2009; 27:451–83. 10.1146/annurev.immunol.021908.132532 19105661

[33] Gallagher KA, Joshi A, Carson WF, Schaller M, Allen R, Mukerjee S, Kittan N, Feldman EL, Henke PK, Hogaboam C, Burant CF, Kunkel SL. Epigenetic changes in bone marrow progenitor cells influence the inflammatory phenotype and alter wound healing in type 2 diabetes. Diabetes. 2015; 64:1420–30. 10.2337/db14-0872 25368099 PMC4375075

[34] Benoit M, Desnues B, Mege JL. Macrophage polarization in bacterial infections. J Immunol. 2008; 181:3733–39. 10.4049/jimmunol.181.6.3733 18768823

[35] Loots MA, Lamme EN, Zeegelaar J, Mekkes JR, Bos JD, Middelkoop E. Differences in cellular infiltrate and extracellular matrix of chronic diabetic and venous ulcers versus acute wounds. J Invest Dermatol. 1998; 111:850–57. 10.1046/j.1523-1747.1998.00381.x 9804349

[36] Yan J, Tie G, Wang S, Tutto A, DeMarco N, Khair L, Fazzio TG, Messina LM. Diabetes impairs wound healing by Dnmt1-dependent dysregulation of hematopoietic stem cells differentiation towards macrophages. Nat Commun. 2018; 9:33. 10.1038/s41467-017-02425-z 29295997 PMC5750226

[37] Mirza RE, Fang MM, Ennis WJ, Koh TJ. Blocking interleukin-1β induces a healing-associated wound macrophage phenotype and improves healing in type 2 diabetes. Diabetes. 2013; 62:2579–87. 10.2337/db12-1450 23493576 PMC3712034

[38] Rachman H, Kim N, Ulrichs T, Baumann S, Pradl L, Nasser Eddine A, Bild M, Rother M, Kuban RJ, Lee JS, Hurwitz R, Brinkmann V, Kosmiadi GA, Kaufmann SH. Critical role of methylglyoxal and AGE in mycobacteria-induced macrophage apoptosis and activation. PLoS One. 2006; 1:e29. 10.1371/journal.pone.0000029 17183656 PMC1762319

[39] Chakrabarti A, Talukdar D, Pal A, Ray M. Immunomodulation of macrophages by methylglyoxal conjugated with chitosan nanoparticles against Sarcoma-180 tumor in mice. Cell Immunol. 2014; 287:27–35. 10.1016/j.cellimm.2013.11.006 24368179

[40] Pal A, Bhattacharya I, Bhattacharya K, Mandal C, Ray M. Methylglyoxal induced activation of murine peritoneal macrophages and surface markers of T lymphocytes in sarcoma-180 bearing mice: involvement of MAP kinase, NF-kappa beta signal transduction pathway. Mol Immunol. 2009; 46:2039–44. 10.1016/j.molimm.2009.03.014 19375802

[41] Rosenstock P, Bezold V, Bork K, Scheffler J, Horstkorte R. Glycation interferes with natural killer cell function. Mech Ageing Dev. 2019; 178:64–71. 10.1016/j.mad.2019.01.006 30659859

[42] Weiskopf D, Weinberger B, Grubeck-Loebenstein B. The aging of the immune system. Transpl Int. 2009; 22:1041–50. 10.1111/j.1432-2277.2009.00927.x 19624493

[43] Montecino-Rodriguez E, Berent-Maoz B, Dorshkind K. Causes, consequences, and reversal of immune system aging. J Clin Invest. 2013; 123:958–65. 10.1172/JCI64096 23454758 PMC3582124

[44] Dinarello CA. Interleukin-1 beta, interleukin-18, and the interleukin-1 beta converting enzyme. Ann N Y Acad Sci. 1998; 856:1–11. 10.1111/j.1749-6632.1998.tb08307.x 9917859

[45] Boucher D, Monteleone M, Coll RC, Chen KW, Ross CM, Teo JL, Gomez GA, Holley CL, Bierschenk D, Stacey KJ, Yap AS, Bezbradica JS, Schroder K. Caspase-1 self-cleavage is an intrinsic mechanism to terminate inflammasome activity. J Exp Med. 2018; 215:827–40. 10.1084/jem.20172222 29432122 PMC5839769

[46] Franchi L, Eigenbrod T, Muñoz-Planillo R, Nuñez G. The inflammasome: a caspase-1-activation platform that regulates immune responses and disease pathogenesis. Nat Immunol. 2009; 10:241–47. 10.1038/ni.1703 19221555 PMC2820724

[47] Brat DJ, Bellail AC, Van Meir EG. The role of interleukin-8 and its receptors in gliomagenesis and tumoral angiogenesis. Neuro Oncol. 2005; 7:122–33. 10.1215/S1152851704001061 15831231 PMC1871893

[48] Harada A, Sekido N, Akahoshi T, Wada T, Mukaida N, Matsushima K. Essential involvement of interleukin-8 (IL-8) in acute inflammation. J Leukoc Biol. 1994; 56:559–64. 10.1002/jlb.56.5.559 7964163

[49] Italiani P, Mazza EM, Lucchesi D, Cifola I, Gemelli C, Grande A, Battaglia C, Bicciato S, Boraschi D. Transcriptomic profiling of the development of the inflammatory response in human monocytes in vitro. PLoS One. 2014; 9:e87680. 10.1371/journal.pone.0087680 24498352 PMC3912012

[50] Tarique AA, Logan J, Thomas E, Holt PG, Sly PD, Fantino E. Phenotypic, functional, and plasticity features of classical and alternatively activated human macrophages. Am J Respir Cell Mol Biol. 2015; 53:676–88. 10.1165/rcmb.2015-0012OC 25870903

[51] Tracey KJ, Cerami A. Tumor necrosis factor: a pleiotropic cytokine and therapeutic target. Annu Rev Med. 1994; 45:491–503. 10.1146/annurev.med.45.1.491 8198398

[52] Spits H, de Waal Malefyt R. Functional characterization of human IL-10. Int Arch Allergy Immunol. 1992; 99:8–15. 10.1159/000236329 1336421

[53] Fiorentino DF, Bond MW, Mosmann TR. Two types of mouse T helper cell. IV. Th2 clones secrete a factor that inhibits cytokine production by Th1 clones. J Exp Med. 1989; 170:2081–95. 10.1084/jem.170.6.2081 2531194 PMC2189521

[54] Villalta SA, Rinaldi C, Deng B, Liu G, Fedor B, Tidball JG. Interleukin-10 reduces the pathology of mdx muscular dystrophy by deactivating M1 macrophages and modulating macrophage phenotype. Hum Mol Genet. 2011; 20:790–805. 10.1093/hmg/ddq523 21118895 PMC3024048

[55] Janeway CA Jr, Medzhitov R. Innate immune recognition. Annu Rev Immunol. 2002; 20:197–216. 10.1146/annurev.immunol.20.083001.084359 11861602

[56] Takeda K, Kaisho T, Akira S. Toll-like receptors. Annu Rev Immunol. 2003; 21:335–76. 10.1146/annurev.immunol.21.120601.141126 12524386

[57] Stuart LM, Ezekowitz RA. Phagocytosis and comparative innate immunity: learning on the fly. Nat Rev Immunol. 2008; 8:131–41. 10.1038/nri2240 18219310

[58] Kierdorf K, Fritz G. RAGE regulation and signaling in inflammation and beyond. J Leukoc Biol. 2013; 94:55–68. 10.1189/jlb.1012519 23543766

[59] Ding AH, Nathan CF, Stuehr DJ. Release of reactive nitrogen intermediates and reactive oxygen intermediates from mouse peritoneal macrophages. Comparison of activating cytokines and evidence for independent production. J Immunol. 1988; 141:2407–12. 3139757

[60] Martinez FO, Gordon S, Locati M, Mantovani A. Transcriptional profiling of the human monocyte-to-macrophage differentiation and polarization: new molecules and patterns of gene expression. J Immunol. 2006; 177:7303–11. 10.4049/jimmunol.177.10.7303 17082649

[61] Sica A, Schioppa T, Mantovani A, Allavena P. Tumour-associated macrophages are a distinct M2 polarised population promoting tumour progression: potential targets of anti-cancer therapy. Eur J Cancer. 2006; 42:717–27. 10.1016/j.ejca.2006.01.003 16520032

[62] Chanput W, Mes J, Vreeburg RA, Savelkoul HF, Wichers HJ. Transcription profiles of LPS-stimulated THP-1 monocytes and macrophages: a tool to study inflammation modulating effects of food-derived compounds. Food Funct. 2010; 1:254–61. 10.1039/c0fo00113a 21776474

[63] Forbes JM, Yee LT, Thallas V, Lassila M, Candido R, Jandeleit-Dahm KA, Thomas MC, Burns WC, Deemer EK, Thorpe SR, Cooper ME, Allen TJ. Advanced glycation end product interventions reduce diabetes-accelerated atherosclerosis. Diabetes. 2004; 53:1813–23. 10.2337/diabetes.53.7.1813 15220206

