# Supplementary material for: Glycation of macrophages induces expression of pro-inflammatory cytokines and reduces phagocytic efficiency
Source: Aging (Albany NY). 2019 Jul 29;11(14):5258–75. doi: 10.18632/aging.102123 (PMC6682540; doi:10.18632/aging.102123)
Supplement: Supplementary Figures [file aging-11-102123-s001.pdf]

SUPPLEMENTARY MATERIAL

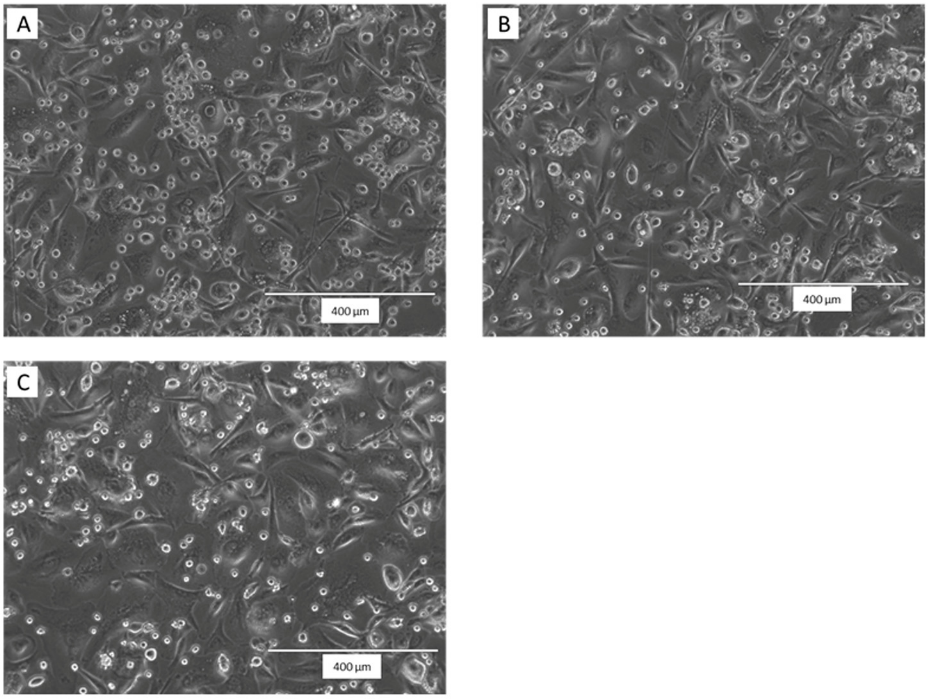

Supplementary Figure 1. Micrographs of macrophages after glycation. Bright field microscopy of THP-1 macrophages (M0) was done after incubation with different MGO concentrations for 24 h in culture medium. (**A**) control; (**B**) 0.5 mM; (**C**) 1 mM. Scale bar indicates 400 µm. Representative micrographs of three different experiments.

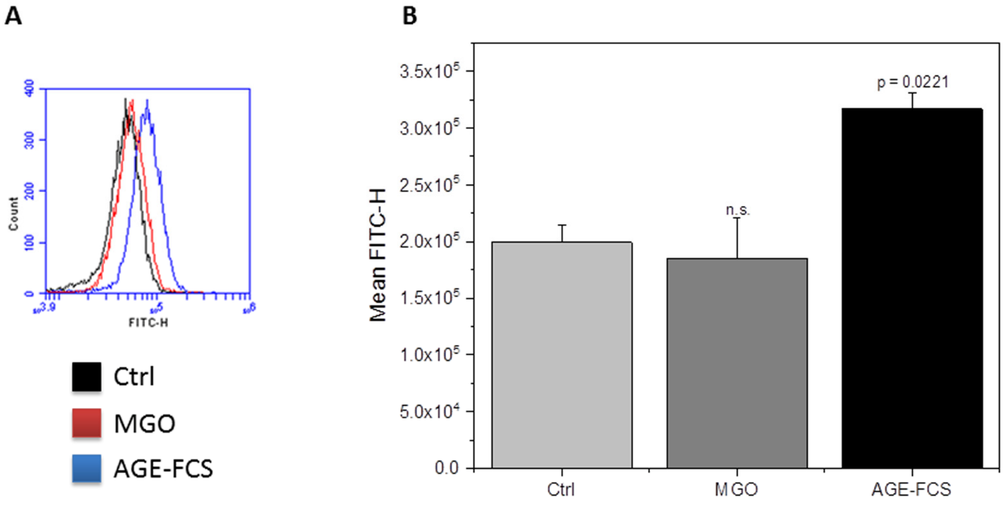

Supplementary Figure 2. Flow cytometry staining of RAGE. THP-1 macrophages (M0) were incubated with 1 mM MGO or 10 % AGE-FCS for 24 h in normal growth medium. Living cells were stained with an anti-RAGE antibody (ab3611) and secondary FITC labeled antibody and analyzed using flow cytometry. (**A**) Representative histogram of analyzed FITC positive macrophages. (**B**) Graph of mean fluorescence intensity of stained macrophages, data represents mean + SD of 3 independent experiments.

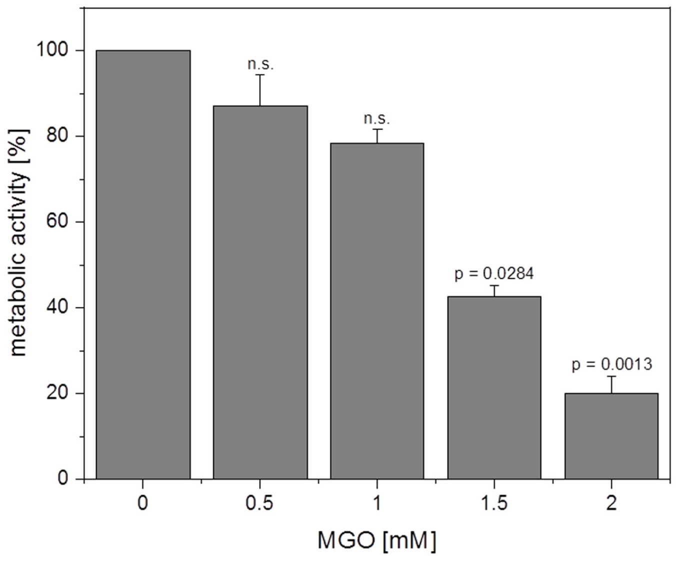

Supplementary Figure 3. Metabolic activity after glycation. THP-1 macrophages (M0) were treated with different concentrations of MGO for 24 h in normal growth medium and MTT assay was performed. Cells without MGO treatment were set to 100 % of metabolic activity. Data represents average mean of metabolic activity + SD of 4 independent experiments.

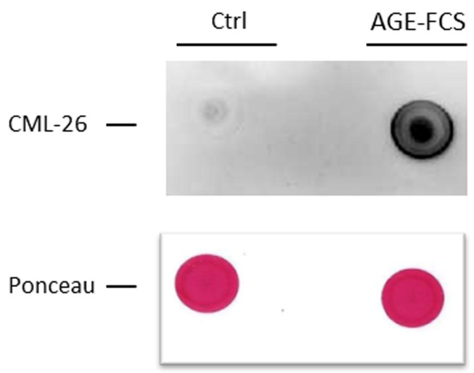

Supplementary Figure 4. Glycation of AGE-FCS. Glycation of AGE-FCS was verified via dot blot using an anti-AGE antibody (CML-26). Shown blot is representative for 3 independent experiments.
